# PstSCAB and SapBCDF are putrescine exporters in *Proteus mirabilis*

**DOI:** 10.1128/spectrum.04306-23

**Published:** 2025-11-14

**Authors:** Yuta Sugiyama, Atsuo Nakamura, Hirokazu Ohta, Tsuyoshi Fujita, Yuki Kontani, Hiromi Shimokawa, Rika Hirano, Yuta Ami, Mikiyasu Sakanaka, Mitsuharu Matsumoto, Shin Kurihara

**Affiliations:** 1 Faculty of Bioresources and Environmental Sciences, Ishikawa Prefectural University 73985, Nonoichi, Ishikawa, Japan; 2 Dairy Science and Technology Institute, Kyodo Milk Industry Co. Ltd 476861 , Hinode, Tokyo, Japan; 3 Faculty of Biology-Oriented Science and Technology, Kindai University 12872, Kinokawa, Wakayama, Japan; Emory University School of Medicine, Atlanta, Georgia, USA

**Keywords:** polyamines, putrescine, *Proteus mirabilis*, swarming, ABC transporters, urinary tract infection

## Abstract

*Proteus mirabilis* is a cause of urinary tract infection. Putrescine, an aliphatic amine, acts as a messenger molecule during *P. mirabilis* urothelial invasion. Inhibition of putrescine-mediated cell-cell communication in *P. mirabilis* may be an effective method for control of urinary tract infection. PlaP is a primary putrescine importer in *P. mirabilis* PM7002. However, no putrescine exporters have been identified to date. In this study, we screened the putrescine exporter gene from *P. mirabilis* PM7002 transposon mutant library using a previously developed high-throughput putrescine quantification method and revealed that PstSCAB is a novel putrescine exporter. SapBCDF and PotE have been reported as a putrescine exporter and putrescine-ornithine antiporter, respectively, in *Escherichia coli*. Although *P. mirabilis* possesses the SapBCDF and PotE homologs, the effects of these homologs on the putrescine export are unclear. We investigated the contributions of SapBCDF and PotE homologs on putrescine export in *P. mirabilis* using gene deletion and found that SapBCDF is a putrescine exporter. *P. mirabilis* exhibits unique and highly coordinated multicellular migration called swarming. Putrescine mediates cell-cell communication during swarming. Therefore, we evaluated the effects of PstSCAB and SapBCDF on swarming. As a result, the swarming diameters of *P. mirabilis* Δ*pstSCAB* and Δ*sapBCDF* were significantly lower than that of the wild type. This revealed that PstSCAB and SapBCDF are putrescine exporters that mediate cell-cell communication during swarming. Inhibition of PstSCAB and SapBCDF is a possible target for treating *P. mirabilis*-related urinary tract infection.

**IMPORTANCE:**

Antibiotics are the primary treatment for bacterial urinary tract infections (UTIs). However, overuse of antibiotics promotes the emergence of antibiotic-resistant bacteria; therefore, it is desirable to develop drugs that specifically inhibit virulence without promoting the emergence of antibiotic resistance. *Proteus mirabilis* causes UTI and uniquely uses putrescine for cell-cell communication during urinary tract invasion. Inhibition of putrescine-mediated cell-cell communication in *P. mirabilis* impairs its invasion of urothelial cells. Therefore, proteins responsible for putrescine-mediated cell-cell communication are a potential target for treating *P. mirabilis* UTIs. However, the putrescine exporter of *P. mirabilis* has yet to be identified. Here, we identified two putrescine exporters of *P. mirabilis*, PstSCAB and SapBCDF. Furthermore, *P. mirabilis* ΔpstSCAB and ΔsapBCDF strains showed a lack of swarming activity. This study indicates PstSCAB and SapBCDF as potential targets for treating UTIs caused by *P. mirabilis.*

## INTRODUCTION

 Antibiotics are an effective treatment for infectious diseases such as urinary tract infections (UTIs). Currently, various antibiotics are used to eliminate pathogens by inhibiting cellular events, such as cell wall synthesis, protein biosynthesis, and nucleic acid synthesis (1). However, overuse of antibiotics accelerates the emergence of antibiotic-resistant bacteria such as methicillin-resistant *Staphylococcus aureus* and vancomycin-resistant *Enterococcus faecalis* (2). Infectious diseases caused by antibiotic-resistant bacteria are difficult to treat with conventional antibiotics. The regulation of virulence factor expression is an effective strategy for eliminating virulent pathogens and reducing the emergence of antibiotic-resistant bacteria. To achieve this, it is essential to identify the mechanisms of virulence factor expression. However, these mechanisms remain unclear in several pathogenic bacteria.

*Proteus mirabilis* is a Gram-negative bacterium that causes UTIs (3). Patients with indwelling urinary catheters have an increased risk of developing bacterial UTIs. Severe UTIs can cause urinary tract stones and sepsis. Therefore, patients with UTIs require appropriate treatment.

*P. mirabilis* performs a highly coordinated multicellular migration, termed swarming, on solid media (4). Swarming involves a complex differentiation cycle between the vegetative and swarmer cells (4). Flagella are involved in various behaviors such as biofilm formation and dispersal (5), swimming (6), and adherence (7). Several studies have shown that flagella contribute to urinary tract colonization and the invasion of host urinary epithelial cells (8, 9). The number of flagella increases 10- to 40-fold during differentiating from vegetative to swarmer cells (10). Similarly, the expression levels of urease and hemolysin genes, which are thought to be virulence factors, are upregulated in swarmer cells (11, 12). Therefore, understanding the mechanism of swarmer cell differentiation and the development of inhibitors of this mechanism could lead to the development of drugs that inhibit UTIs.

 Putrescine is a signaling molecule involved in cell-to-cell communication during *P. mirabilis* swarming and urothelial invasion (13). *P. mirabilis* differentiates into swarmer cells by sensing extracellular putrescine (13). Extracellular putrescine concentration is presumed to be regulated by putrescine export and uptake by *P. mirabilis*. PlaP has been identified as a putrescine importer in *P. mirabilis* (14); however, no putrescine exporters have previously been identified. In contrast, in *Escherichia coli*, which belongs to the same order *Enterobacterales* as *P. mirabilis*, the putrescine exporter SapBCDF (15) and putrescine-ornithine antiporter PotE (16) have been identified. In *P. mirabilis*, *sapD* is encoded in the *sapABCDF* operon and a transposon insertion in *sapD* shows antimicrobial sensitivity, and SapABCDF has been reported to import antimicrobial peptide into the intracellular space and contribute to antimicrobial resistance (17). Although the genes encoding SapBCDF and PotE homologs are present in the genome of *P. mirabilis*, the function of these proteins in putrescine export in *P. mirabilis* is still unclear.

 Transposon mutagenesis and comprehensive phenotypic analysis are useful for analyzing the function of genes and obtaining mutants that exhibit the desired phenotype. A transposon mutagenesis method has been established for *Proteus* species (18–20), and it is possible to search for mutants that exhibit the desired phenotype, such as failure to export putrescine to the culture supernatant. However, high-throughput quantification of putrescine in the culture supernatant of many bacterial strains is difficult because of cumbersome processes such as derivatization and time-consuming analysis using high-performance liquid chromatography (HPLC) (21) or gas chromatography (GC) (22). We recently developed a high-throughput method to quantify putrescine using putrescine oxidase (PuO) in a 96-well plate (PuO-POD-4AA-TOPS method) (23). This system allows high-throughput quantification of putrescine levels in the culture supernatants of *P. mirabilis* without putrescine derivatization (23).

 In this study, the *P. mirabilis* transposon mutant library was screened for unidentified putrescine exporters using putrescine levels in the culture supernatant as an indicator. We found that PstSCAB is a novel putrescine exporter and is required for swarming motility. Furthermore, the swarming motility and putrescine export ability of the gene deletion mutants for the PotE and SapBCDF homologs revealed that the SapBCDF homolog is a putrescine exporter.

## MATERIALS AND METHODS

### Chemicals

Putrescine dihydrochloride was purchased from Tokyo Chemical Industry Co., Ltd. (Tokyo, Japan). Stable isotope-labeled L-arginine-^13^C_6_,^15^N_4_ (S.I.Arg) was obtained from Fujifilm Wako Pure Chemical (Osaka, Japan). All other reagents were of analytical grade.

### Bacterial strain, plasmid, and culture conditions

The bacterial strains and plasmids used in this study are listed in Table 1. *P. mirabilis* was aerobically cultured in Luria-Bertani (LB)-Miller (BD Difco, Franklin Lakes, NJ, USA; 1% tryptone, 1% NaCl, and 0.5% yeast extract) or LB-Lennox medium (1% tryptone, 0.5% NaCl, and 0.5% yeast extract) at 37°C. *E. coli* was aerobically cultured in LB-Miller medium (BD Difco) at 37°C.

**TABLE 1:** Strains and plasmids used in this study

Strain or plasmid	Genotype or description	Reference
*P. mirabilis*		
PM7002	Wild-type	Laboratory stock
PM437	Δ*speA*::mini-Tn5*lacZ1*	(13)
HO110	Δ*potE*::*Cm^R^*	This study
HO118	Δ*sapBCDF*::*Cm^R^*	This study
HO119	Δ*pstSCAB*::*Cm^R^*	This study
KY4	Δ*sapBCDF*::*Cm^R^* Δ*pstSCAB*::*Kan^R^*	This study
TF13	pSK710/PM7002	This study
TF17	pSK710-*pstS*^+^*C*^+^*A*^+^*B*^+^/HO119	This study
TF24	pSK710/HO119	This study
*E. coli*		
CC118 λ*pir*	Δ(*ara-leu*), *araD*, Δ*lacX74*, *galE*, *galK*, *phoA20*, *thi-1*, *rpsE*, *rpoB*, *argE*(Am), *recA1*, λ*pir* lysogen	(24)
SM10 λ*pir*	*thi thr leu tonA lacY supE recA*::RP4-2-Tc::Mu Km*^R^* λ*pir*	(25)
HO103	CC118 λ*pir* harboring pHO103	This study
HO104	CC118 λ*pir* harboring pHO104	This study
HO105	CC118 λ*pir* harboring pHO104	This study
Plasmid		
pKNG101	R6K replicon *mob*^+^ *sacB*^+^*R*^+^ streptomycin resistance gene (*Sm^R^*)	(26)
pKNG101-Δ*plaP*	R6K replicon *mob*^+^ *sacB*^+^*R*^+^ Δ*plaP*::*Cm^R^ Sm^R^*	(14)
pACYC184	p15A replicon *Cm^R^ Tet^R^*	New England Biolabs
pSK710	p15A replicon *Cm^R^ Sm^R^*	This study
pSK710-*pstS*^+^*C*^+^*A*^+^*B*^+^	p15A replicon *pstS*^+^*C*^+^*A*^+^*B*^+^ *Cm^R^ Sm^R^*	This study
pHO103	R6K replicon *mob*^+^ *sacB*^+^*R*^+^ Δ*potE*::*Cm^R^ Sm^R^*	This study
pHO104	R6K replicon *mob*^+^ *sacB*^+^*R*^+^ Δ*sapBCDF*::*Cm^R^ Sm^R^*	This study
pHO105	R6K replicon *mob*^+^ *sacB*^+^*R*^+^ Δ*pstSCAB*::*Cm^R^ Sm^R^*	This study
pKY1	R6K replicon *mob*^+^ *sacB*^+^*R*^+^ Δ*pstSCAB*::*Kan^R^ Sm^R^*	This study

### Construction of *P. mirabilis* transposon mutant library

The cells from 15 mL culture with optical density at 600 nm (OD_600_) of 0.4 were collected by centrifugation (3,000 *× g*, 4°C, 5 min), washed twice with 10% (vol/vol) glycerol solution, and then resuspended in 60 µL of 10% glycerol solution. A transposon mutant library was constructed using EZ-Tn5 <KAN-2> Tnp Transposome Kit (Epicentre Technologies, Madison, WI, USA) according to the manufacturer’s protocol. Transposon-insertion mutants were selected on LB-Lennox agar medium containing 20 µg/mL kanamycin and 15 µg/mL tetracycline, and a total of 1,248 colonies were obtained. The colonies were used as transposon-insertion mutants. Each transposon-insertion mutant was inoculated into 500 µL of LB-Lennox broth containing 20 µg/mL kanamycin and 15 µg/mL tetracycline in a 96-well deep-well plate and cultured for 24 h at 37°C.

### Enzymatic evaluation of putrescine concentration in the culture supernatant of *P. mirabilis* transposon mutants

Each transposon-insertion mutant was grown in 500 µL of LB-Lennox broth containing 20 µg/mL kanamycin and 15 µg/mL tetracycline in a 96-well deep-well plate and cultured for 24 h at 37°C. One hundred microliters of culture was mixed with 100 µL of 1 × phosphate-buffered saline (PBS), and OD_600_ was measured using a Multiskan GO Microplate Spectrophotometer (Thermo-Fisher, Waltham, MA, USA). The plates were centrifuged (2,500 *× g*, 4°C, 15 min), and the supernatants were collected and stored at −20°C until use. Assays were performed using the PuO-POD-4AA-TOPS method as described previously (23) with minor modifications. Briefly, 200 µL of the reaction mixture containing 50 mM Tris-HCl (pH 8.0), 1 mM *N*-ethyl-*N*-(3-sulfopropyl)-3-methylaniline, 1 mM 4-aminoantipyrin, 5 U/mL horseradish peroxidase, 0.1 µg/mL PuO, and 10 µL culture supernatant was incubated on 96-well plate at 25°C for 10 min, and the absorbance at 550 nm (*A*_550_) was continuously monitored. The *A*_550_ value after 10 min of incubation was used to evaluate the putrescine concentration. A standard curve was generated from a putrescine solution of known concentration. Putrescine concentrations determined by the assay were normalized by the OD_600_ value, and the normalized values were referred to as the Put level (μM/OD_600_).

### Quantification of putrescine using high-performance liquid chromatography

 Culture supernatants were collected by centrifugation (18,700 *× g*, 4°C, 5 min) and diluted 10-fold with Milli-Q water and then treated with 1/10th volume of 100% (wt/vol) trichloroacetic acid (TCA) and centrifuged (18,700 *× g*, 4°C, 10 min) to remove proteins. The supernatants were filtered using Cosmonice filter W (Nacalai Tesque, Kyoto, Japan), and the filtrates were subjected to HPLC analysis. The cell pellets were resuspended in 300 µL of 5% (wt/vol) TCA and boiled for 15 min to disrupt the cells. The suspension was centrifuged (18,700 *× g*, 4°C, 10 min) to remove cell debris, the supernatants were filtered through Cosmonice filter W, and the filtrates were analyzed by HPLC. The cell debris was dissolved in 300 µL of 0.1 *N* NaOH, and the protein concentration was measured using a protein assay kit (Bio-Rad, Hercules, CA, USA). The HPLC analytical conditions were the same as those described previously (27). The putrescine concentrations in the culture supernatants were normalized by OD_600_ value and indicated as μM/OD_600_. Intracellular putrescine levels were normalized by the total cellular protein and expressed as nmol/mg protein.

### Determination of the transposon insertion site

 The transposon insertion site was determined by a two-step semi-degenerate PCR with reference to a previous study (28). The primers and primer pairs used in this study are listed in Tables S1 and S2, respectively. Genomic DNA (gDNA) of the mutant was extracted and purified using a Wizard Genome DNA Purification Kit (Promega, Madison, WI, USA). PrimeSTAR GXL (Takara Bio, Kusatsu, Japan) was used for the PCR. First-step PCR was performed using the primer pairs Pr1/Pr2 or Pr1/Pr3 with gDNA as a template. The PCR product was purified and used as a template in the second-step PCR with primer pairs Pr4/Pr5 and Pr4/Pr6. The DNA fragment from the second-step PCR was purified and analyzed using primers Pr7 and Pr8. The sequence results were subjected to BLASTn analysis (29) using *P. mirabilis* HI4320 (taxid:529507) as the query sequence.

### Deletion of *pstSCAB***,** *sapBCDF*, and *potE*

 Gene deletion of *pstSCAB*, *sapBCDF*, and *potE* was performed using pKNG101, as reported previously (14). The primers and primer pairs are listed in Tables S1 and S2. *E. coli* CC118 λ*pir* was used for plasmid construction. Five hundred base pairs upstream and downstream of the target gene were amplified using KOD Plus Neo (Toyobo, Osaka, Japan) with the gDNA of *P. mirabilis* PM7002 as a template. The chloramphenicol-resistance gene (*Cm^R^*) was amplified from pKNG101 (26) and ligated with 500 bp downstream of the target gene using PCR ligation. Then, the DNA fragment obtained by PCR ligation was ligated with 500 bp upstream of the target gene using PCR ligation to generate DNA fragments with the *Cm^R^* sandwiched between the upstream and downstream regions of the target gene. The generated DNA fragment was inserted into the SmaI site of pKNG101 using the In-Fusion HD Cloning Kit (Takara Bio). The resulting plasmid was introduced into *P. mirabilis* PM7002 by mating using *E. coli* SM10 λpir as described below. Cells from 1 mL of overnight culture of *P. mirabilis* PM7002 and *E. coli* SM10 harboring pKNG101-Δ*potE::Cm^R^* (HO103), pKNG101-Δ*sapBCDF::Cm^R^* (HO104), or pKNG101-Δ*pstSCAB::Cm^R^* (HO105) were collected by centrifugation and washed twice with fresh LB-Miller broth. The washed cells were resuspended in 1 mL of fresh LB-Miller broth, and 100 µL of *P. mirabilis* PM7002 suspension was mixed with 100 µL of HO103, HO104, or HO105 suspension. The mixture was spread on an LB-Lennox agar plate and incubated for 8 h at 37°C. After incubation, the cells grown on LB-Lennox agar plates were collected and suspended in 4 mL of fresh LB-Lennox broth. One hundred microliters of suspension was spread on LB-Lennox agar plate containing 35 µg/mL chloramphenicol and 15 µg/mL tetracycline and then incubated overnight at 37°C. Insertion of the plasmid into the *P. mirabilis* chromosome was confirmed by colony PCR. pKNG101 possesses the *sacB* gene, which encodes levansucrase and is cytotoxic under high sucrose conditions (26). Therefore, in order to screen the bacterial strains for second cross-over events, the plasmid-insertion mutants were cultured overnight in LB-Miller broth at 37°C with shaking at 140 rpm. The overnight culture was serially diluted with sterilized PBS and spread on LB-Miller agar plates containing 35 µg/mL chloramphenicol and 10% (wt/vol) sucrose and incubated for 31 h at 37°C. The genotypes of the grown colonies were checked by colony PCR. The construction of a double-knockout strain (Δ*sapBCDF* D*pstSCAB*) of candidate transporter genes was performed as follows: A kanamycin-resistance gene (*Kan^R^*) was amplified from the gDNA of HO93 (*pstS*::EZ-Tn5 [Kan2]). Amplified *Kan^R^* was ligated with 500 bp of the downstream of *pstSCAB* using PCR ligation, and the amplified PCR fragment was ligated with 500 bp of the upstream of *pstSCAB* using PCR ligation to generate DNA fragments with the *Kan^R^* gene sandwiched between the upstream and downstream regions of *pstSCAB*, and cloned into SmaI site of pKNG101 using the In-Fusion HD Cloning Kit (Takara Bio). The resulting plasmid pKY1 was introduced into HO118 (Δ*sapBCDF*) by mating using *E. coli* SM10 λpir. Plasmid insertion and genotype analyses were performed as described above. The genomes of the deletion mutants were sequenced to confirm the absence of undesired mutations.

### Complementation of *pstSCAB*

 Complementation of *pstSCAB* was carried out using pSK710. The streptomycin resistance gene (*Sm^R^*) was excised from pKNG101 by digestion with BamHI and EcoRV. The *Sm^R^* fragment was inserted into the BamHI and HincII sites of pACYC184, resulting in plasmid pSK710. The DNA fragment containing *pstSCAB* and 500 bp upstream of *pstS* was chemically synthesized and cloned into HindIII and SphI sites of pSK710 by GenScript (NJ, US). The plasmids were introduced into *P. mirabilis* by electroporation.

### Quantification of phosphate in the culture supernatants

 *P. mirabilis* WT and HO119 (Δ*pstSCAB*) were streaked on LB-Miller agar plates (3% agar) and cultured at 37°C overnight, then a single colony was inoculated in 5 mL of LB-Miller medium and further cultured overnight at 37°C with shaking at 140 rpm. Ten microliters of culture was inoculated into 100 mL LB-Lennox medium and cultivated at 37°C with shaking at 140 rpm. The cultures were collected and centrifuged (19,397 *× g*, 4°C, 5 min), and the supernatants were collected and used for phosphate quantification. Phosphate in the culture supernatants was quantified using PiBlue Phosphate Assay Kit (BioAssay Systems, Hayward, CA).

### Swarm assays

The tested strains were streaked on LB-Miller agar plates (3% agar) and cultured at 37°C overnight. A single colony was inoculated in 5 mL of LB-Miller medium and further cultured overnight at 37°C with shaking at 140 rpm. Two microliters of overnight culture were spotted on an LB-Miller agar plate (1.5% agar) with or without 50 µM putrescine, and the plate surface was allowed to dry by leaving the lid of the dish open for 5 min. Then, the plates were incubated at 37°C. The swarming diameter was measured at the indicated time points. A photograph of swarming was taken after 21 h of incubation using a LAS-3000 Luminescent Image Analyzer (Fujifilm, Tokyo, Japan) or a single lens reflex camera D5500 (Nikon, Tokyo, Japan).

### Swim assay

 The tested strains were streaked on LB-Miller agar plates (3% agar) and cultured at 37°C overnight. Then, a single colony was inoculated in 5 mL of LB-Miller medium and cultured overnight at 37°C with shaking at 140 rpm. A sterile toothpick tip was immersed in the culture medium, inoculated by vertically puncturing the center of the LB-Miller agar plate (agar 0.25%), and incubated at 37°C. After 13 h of incubation, the swarming diameter was measured and a photograph was taken using an iPhone 15 (Apple, CA, USA).

### Quantification of stable isotope-labeled putrescine using gas chromatography-mass spectrometry

 *Proteus mirabilis* PM7002 (wild-type, WT), HO118 (Δ*sapBCDF*), HO119 (Δ*pstSCAB*), and KY4 (Δs*apBCDF* Δ*pstSCAB*) were streaked on LB-Miller agar plates (3% agar) and cultured at 37°C overnight. A single colony was inoculated into LB-Miller medium and pre-cultured overnight at 37°C with shaking at 140 rpm. The pre-culture was inoculated into 10 mL of M9 + Tryptone medium containing 3 mM S.I.Arg to obtain an OD_600_ of 0.03 and further cultured for 8 h at 37°C with shaking at 140 rpm. The culture was centrifuged (19,397 *× g*, 4°C, 2 min), and the supernatants were collected. Five hundred microliters of TCA was added to 5 mL of the culture supernatants and centrifuged (19,397 *× g*, 4°C, 10 min). The supernatants were collected and analyzed using GC-MS (GCMS-QP2010, Shimadzu, Kyoto, Japan).

 Putrescine derivatization was performed as described previously using ethyl chloroformate and trifluoroacetic anhydride (15), with 1,6-diaminohexane as the internal standard.

### Statistical analysis

Statistical analyses were performed using BellCurve for Excel (Social Survey Research Information Co., Ltd., Tokyo, Japan) and SPSS version 28 (IBM Corp., Armonk, NY, USA). Statistical significance was assessed using Student’s *t*-test, or one-way analysis of variance (ANOVA), followed by Tukey’s test for multiple comparisons, and *P* values < 0.05 were considered statistically significant. Data were reported as the mean ± standard deviation (SD).

## RESULTS

### Screening for gene encoding putrescine exporter

**Fig 1:**
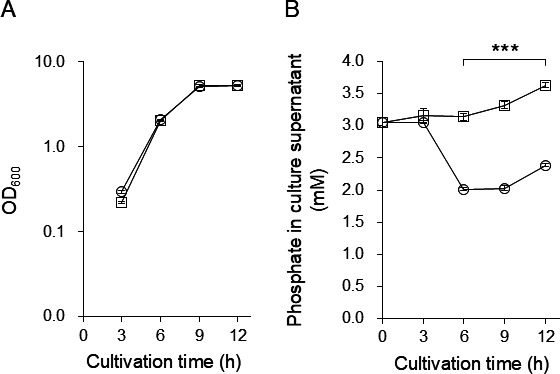
Effect of *pstSCAB* deletion on phosphate concentration in culture supernatant. *Proteus mirabilis* PM7002 (WT) and HO119 (Δ*pstSCAB*) were grown in LB-Miller broth. Phosphate concentrations in culture supernatants and OD_600_ measurements were taken at the indicated time points. Experiments were performed in triplicate for each strain and the mean ± SD are shown. (**A**) Growth curve of *P. mirabilis* PM7002 (WT) and HO119 (Δ*pstSCAB*). White circles and squares indicate OD_600_ values for WT and Δ*pstSCAB*, respectively. (**B**) Phosphate concentration in culture supernatants of *P. mirabilis* PM7002 (WT) and HO119 (Δ*pstSCAB*). White circles and squares indicate phosphate concentrations in culture supernatants of WT and Δ*pstSCAB*, respectively. Statistical significance was assessed using one-way ANOVA, followed by Tukey’s test. Means with different letters are significantly different (*P* < 0.05).

 We screened for strains with low putrescine concentrations in the culture supernatant based on the hypothesis that the putrescine concentration in the culture supernatant of strains in which the gene encoding the putrescine exporter is disrupted would be lower than that of the WT. The putrescine concentration in the culture supernatants of 1,246 *P*. *mirabilis* transposon-insertion mutants was measured using the PuO-POD-4AA-TOPS method (23). As putrescine concentrations in culture supernatants are partially dependent on cell growth (15), the putrescine concentration of each strain was normalized by the OD_600_ value. A total of 25 strains were identified as putrescine exporter gene-deletion candidates after comparison with the WT (Fig. S1A). Putrescine concentrations in the culture supernatants of these candidates were analyzed in more detail by HPLC, and the concentration of putrescine in the culture supernatant of strains 1–18, 2–37, 4–72, and 5–13 was over 20% lower than that of WT (Fig. S1). Next, the transposon insertion site of strains 1–18, 2–37, 4–72, and 5–13 was determined by a two-step semi-degenerate PCR. In strains 1–18 and 5–13, transposons were inserted into *PMI2809*, which is annotated to encode methyl-accepting chemotaxis protein (30), and in strain 4–72, a transposon was inserted into *PMI3180*, which is annotated to encode M23B family outer membrane metalloprotease (31). In strain 2–37, a strain with the lowest putrescine concentration in the culture supernatant, a transposon was inserted into *pstS*, which is annotated to encode a solute-binding protein of the phosphate ATP-binding cassette transporter, *pstSCAB*. PstSCAB is a virulence factor of *P. mirabilis* HI4320 (32, 33) and regulates phosphatase activity in phosphate-rich environments (32). Since the objective of this study was to identify transporters, we decided to analyze PstSCAB. *P. mirabilis* possesses a *pst* operon with similarity in the range of 77%‒91% to other bacterial species, such as *E. coli*, *Haemophilus influenzae*, and *Salmonella* Typhimurium (Table S3). PstSCAB has been reported as an inorganic phosphate transporter in several bacteria such as *E. coli* (34). However, the contribution of PstSCAB on phosphate transport has not been reported in *P. mirabilis*. First, the function of PstSCAB on phosphate uptake was evaluated using the Δ*pstSCAB* strain. There were no changes in growth due to *pstSCAB* deletion (Fig. 1A). In the wild type, the phosphate in the culture supernatants was decreased concomitantly with cell growth. In contrast, no such decrease was observed in Δ*pstSCAB* (Fig. 1B). These results indicated that PstSCAB is a phosphate transporter in *P. mirabilis*. We hypothesized that PstSCAB in *P. mirabilis* possesses two functions: as an inorganic phosphate transporter and a putrescine exporter. Two putrescine exporters, PotE and SapBCDF, have been reported in *E. coli* MG1655 (15, 16). Although *P. mirabilis* possesses PotE and SapBCDF homologs (Table S3), the physiological functions of these homologs have not been determined experimentally. Therefore, the functions of PstSCAB, PotE, and SapBCDF in putrescine export in *P. mirabilis* were investigated using Δ*pstSCAB*, Δ*potE*, and Δ*sapBCDF* mutants.

### *pstSCAB* and *sapBCDF* affect the putrescine concentrations in culture supernatants

**Fig 2:**
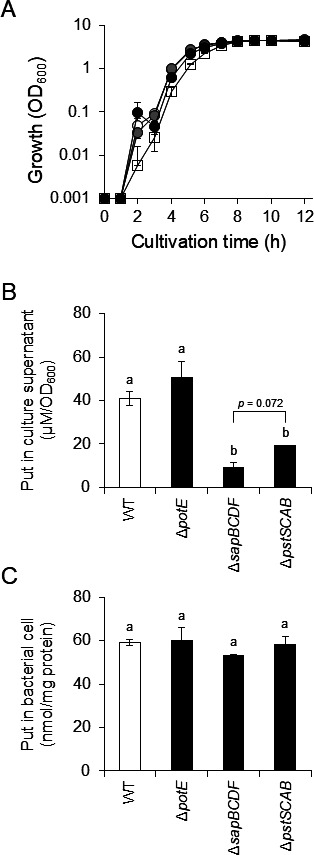
Effect of *potE*, *sapBCDF*, and *pstSCAB* deletion on putrescine concentrations in culture supernatants and intracellular putrescine concentration of *P. mirabilis* PM7002. *Proteus mirabilis* PM7002 (WT), HO110 (Δ*potE*), HO118 (Δ*sapBCDF*), and HO119 (Δ*pstSCAB*) were grown in LB-Miller broth. After 9 h of culture, putrescine concentrations in culture supernatants and cells were measured by HPLC. Experiments were performed in triplicate for each strain and the mean ± SD are shown. (**A**) Growth curve of *P. mirabilis* PM7002 (WT), HO110 (Δ*potE*), HO118 (Δ*sapBCDF*), and HO119 (Δ*pstSCAB*). OD_600_ values were measured at the indicated times. White circles, gray circles, white squares, and black circles indicate OD_600_ of PM7002, HO110, HO118, and HO119, respectively. (**B**) Putrescine concentration in culture supernatant. Putrescine concentrations are normalized by the OD_600_ value and indicated as μM/OD_600_. (**C**) Intracellular putrescine concentration. Putrescine concentrations are normalized by the cellular protein levels and are indicated as nmol/mg protein. Statistical significance was assessed using one-way ANOVA, followed by Tukey’s test. Means with different letters are significantly different (*P* < 0.05).

 *P. mirabilis* Δ*pstSCAB* (HO119), Δ*potE* (HO110), and Δ*sapBCDF* (HO118) strains were generated using double cross-over recombination using pKNG101 (14) (Fig. S3). Delayed growth in the logarithmic phase was observed in HO118 and HO119, whereas HO110 grew in the same manner as PM7002 (WT) (Fig. 2A). Putrescine concentrations in the culture supernatants of *P. mirabilis* PM7002, HO110, HO118, and HO119 were quantified by HPLC after 9 h of culture. The putrescine concentrations in the culture supernatants of HO118 (9.4 ± 1.9 µM/OD_600_) and HO119 (19 ± 0.2 µM/OD_600_) were significantly lower than that of PM7002 (41 ± 3.0 µM/OD_600_), whereas the putrescine concentration in the culture supernatant of HO110 (51 ± 7.4 µM/OD_600_) was the same as that of PM7002 (Fig. 2B). To demonstrate that the decrease in putrescine concentration in the culture supernatant was not due to inhibition of the intracellular putrescine synthesis pathway, intracellular putrescine amounts were measured. No significant differences were observed between the tested strains (Fig. 2C). These results indicated that SapBCDF and PstSCAB are involved in putrescine export, whereas PotE is not.

### Putrescine concentrations in the culture supernatants of *P. mirabilis ΔpstSCAB* Δ*sapBCDF* double-knockout mutant

**Fig 3:**
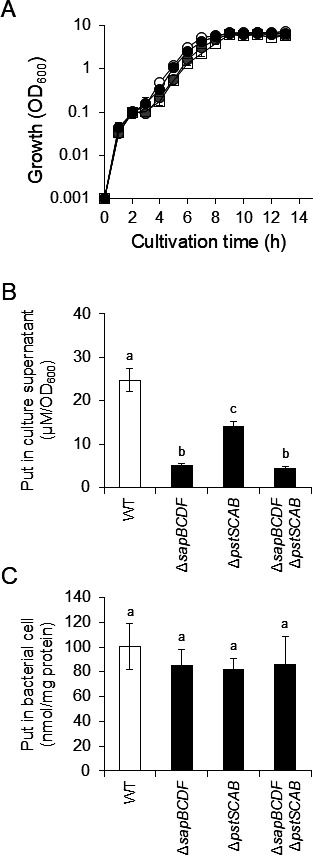
Putrescine concentration in culture supernatants and intracellular putrescine concentration in *P. mirabilis* Δ*sapBCDF* Δ*pstSCAB. P. mirabilis* PM7002 (WT), HO118(Δ*sapBCDF*), HO119 (Δ*pstSCAB*), and KY4 (Δ*sapBCDF* Δ*pstSCAB*) were grown in LB-Lennox broth. After 9 h of culture, putrescine concentrations in culture supernatants and cells were measured by HPLC. Experiments were performed in triplicate for each strain and the mean ± SD are shown. (**A**) Growth curve of *P. mirabilis* PM7002 (WT), HO118(Δ*sapBCDF*), HO119 (Δ*pstSCAB*), and KY4 (Δ*sapBCDF* Δ*pstSCAB*). OD_600_ values were measured at the indicated times. White circles, gray circles, black circles, and white squares indicate OD_600_ of PM7002, HO118, HO119, and KY4, respectively. (**B**) Putrescine concentration in the culture supernatant. Putrescine concentrations are normalized by the OD_600_ value and are indicated as μM/OD_600_. (**C**) Intracellular putrescine concentration. Putrescine concentrations are normalized by the cellular protein levels and are indicated as nmol/mg protein. Statistical significance was assessed using one-way ANOVA followed by Tukey’s test. Means with different letters are significantly different (*P* < 0.05).

 As putrescine was still observed in the culture supernatants of HO118 and HO119 at the concentrations of 9.4 ± 1.9 µM/OD_600_ and 19 ± 0.2 µM/OD_600_, respectively (Fig. 2B), we speculated that if only SapBCDF and PstSCAB were putrescine exporters, deletion of both *sapBCDF* and *pstSCAB* would result in undetectable putrescine in the culture supernatant. Therefore, a *P. mirabilis* Δ*sapBCDF* Δ*pstSCAB* double-knockout strain (KY4) (Fig. S3) was constructed, and putrescine concentrations in the culture supernatants were measured. Although KY4 showed the greatest growth retardation compared to PM7002 (WT), HO118 (Δ*sapBCDF*), and HO119 (Δ*pstSCAB*), the OD_600_ values of PM7002, HO118, HO119, and KY4 were almost the same after 9 h of culture (Fig. 3A). The putrescine concentrations in the culture supernatants and cells were quantified after 9 h of culture. Unexpectedly, putrescine was still observed in the culture supernatants of KY4 (4.4 ± 0.4 µM/OD_600_), and the amounts were same as HO118 (5.2 ± 0.3 µM/OD_600_) (Fig. 3B). However, there was no significant difference in the intracellular putrescine concentrations between the tested strains (Fig. 3C).

### Intracellular putrescine is exported into the culture supernatants through PstSCAB and SapBCDF

**Fig 4:**
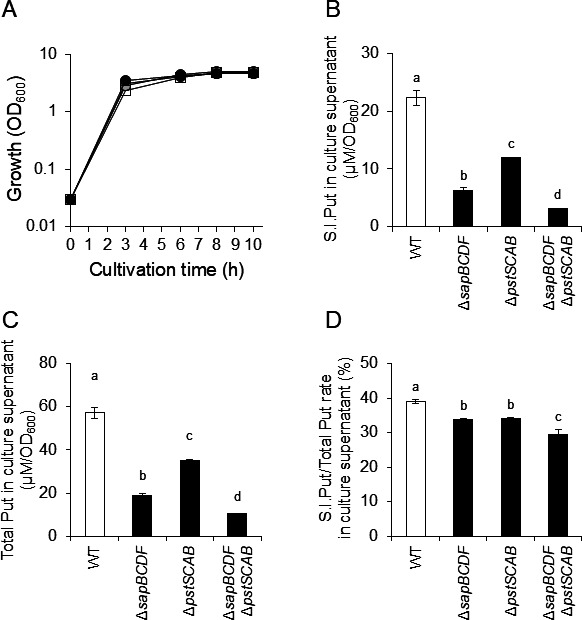
Stable isotope-labeled putrescine in culture supernatants of *P. mirabilis* WT, Δ*pstSCAB*, Δ*sapBCDF*, and Δ*pstSCAB* Δ*sapBCDF. P. mirabilis* PM7002 (WT), HO118 (Δ*sapBCDF*), HO119 (Δ*pstSCAB*), and KY4 (Δ*sapBCDF* Δ*pstSCAB*) were grown in M9 + Tryptone medium containing 3 mM stable isotope-labeled L-arginine (S.I.Arg). After 9 h of culture, putrescine concentrations in culture supernatants were measured using GC-MS. Experiments were performed in biological triplicate and the mean ± SD are shown. Statistical significance was assessed using one-way ANOVA followed by Tukey’s test. Means with different letters are significantly different (*P* < 0.05). (**A**) Growth curve of *P. mirabilis* PM7002 (WT), HO118 (Δ*sapBCDF*), HO119 (Δ*pstSCAB*), and KY4 (Δ*sapBCDF* Δ*pstSCAB*). White circles, gray circles, black circles, and white squares indicate the OD_600_ of PM7002, HO110, HO118, and KY4, respectively. Culture supernatants were collected after 8 h cultivation. (**B**) Stable isotope-labeled putrescine (S.I.Put) in culture supernatants of *P. mirabilis* PM7002 (WT), HO118(Δ*sapBCDF*), HO119 (Δ*pstSCAB*), and KY4 (Δ*sapBCDF* Δ*pstSCAB*). S.I.Put are normalized by the OD_600_ value and indicated as μM/OD_600_. (**C**) Total Put in culture supernatants of *P. mirabilis* PM7002 (WT), HO118(Δ*sapBCDF*), HO119 (Δ*pstSCAB*), and KY4 (Δ*sapBCDF* Δ*pstSCAB*). Total Put is normalized by the OD_600_ value and indicated as μM/OD_600_. (**D**) Ratio of S.I.Put to total Put in culture supernatant.

 To demonstrate that intracellular putrescine was exported into the culture supernatant through PstSCAB and SapBCDF, an assay using stable isotope-labeled arginine (S.I.Arg) was performed. As L-arginine is a putrescine precursor, it is thought that Arg imported into bacterial cells is metabolized sequentially by arginine decarboxylase (SpeA) and agmatine ureohydrolase (SpeB) to stable isotope-labeled putrescine (S.I.Put) (15) (Fig. S4). When 3 mM of L-arginine was added to the medium for culturing *P. mirabilis* PM7002 (WT), the concentration of putrescine in the culture supernatant increased by a mean of 29% at each culture timepoint (Fig. S5). However, putrescine was not detected in the culture supernatant of *P. mirabilis* (Δ*speA*) and adding 3 mM L-arginine did not increase putrescine in the culture supernatant (Fig. S5). Furthermore, there are no previous reports of *P. mirabilis* extracellularly converting L-arginine to putrescine. Altogether, the arginine added to the medium was likely taken up by the *P. mirabilis* cells, subsequently converted to putrescine by SpeA and SpeB, and exported into the culture supernatants. To distinguish the added L-arginine from the L-arginine initially present in the medium and synthesized intracellularly by *P. mirabilis*, S.I.Arg was added to the medium. The concentration of S.I.Put in the culture supernatant of the mutant strain with deleted candidate transporter genes for S.I.Put was compared to that of the WT strain. PM7002 (WT), HO118 (Δ*sapBCDF*), HO119 (Δ*pstSCAB*), and KY4 (Δ*sapBCDF* Δ*pstSCAB*) were cultured in LB-Lennox medium supplemented with S.I.Arg, and the concentrations of S.I.Put in the culture supernatants were measured after 8 h of culture (Fig. 4; Fig. S6). Growth of *P. mirabilis* did not change with arginine supplementation (Fig. S7) and S.I.Put in the culture supernatants of PM7002 (WT) was 22 ± 1.3 µM/OD_600_, whereas HO118 (Δ*sapBCDF*), HO119 (Δ*pstSCAB*), and KY4 (Δ*sapBCDF* Δ*pstSCAB*) were significantly lower, at 6.3 ± 0.4 µM/OD_600_, 12 ± 0.1 µM/OD_600_, and 3.1 ± 0.1 µM/OD_600_, respectively (Fig. 4B). Additionally, S.I.Put level of KY4 (Δ*sapBCDF* Δ*pstSCAB*) was also significantly lower than that of HO118 (Δ*sapBCDF*) and HO119 (Δ*pstSCAB*) (Fig. 4B). Total putrescine concentrations showed similar trends to those of S.I.Put (Fig. 4C). These results indicated that PstSCAB and SapBCDF contribute to intracellular putrescine export to culture supernatants in PM7002. In addition, there were small but significant differences between PM7002 and gene deletion mutants in the ratio of S.I.Put to total putrescine (Fig. 4D).

### Swarming and swimming activity of *P. mirabilis* PM7002 mutants

**Fig 5:**
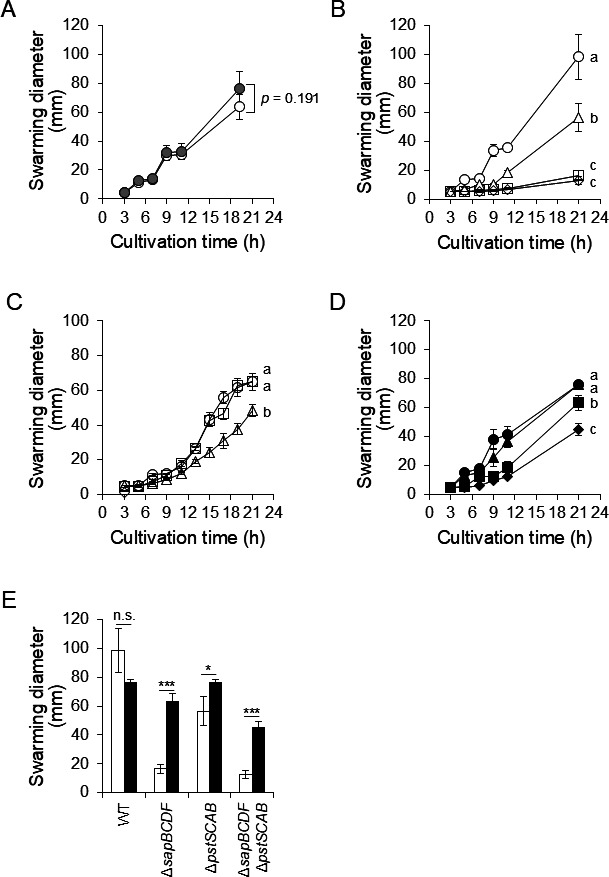
Swarming activity of *P. mirabilis* WT, Δ*pstSCAB*, Δ*sapBCDF*, and Δ*pstSCAB* Δ*sapBCDF. P. mirabilis* PM7002 (WT), HO110 (Δ*potE*) HO118 (Δ*sapBCDF*), HO119 (Δ*pstSCAB*), KY4 (Δ*sapBCDF* Δ*pstSCAB*), TF13 (pSK710/PM7002), TF17 (pSK710-*pstS*^+^*C*^+^*A*^+^*B*^+^/HO119), and TF24 (pSK710/HO119) were grown overnight in LB-Miller broth. Two microliters of overnight cultures was spotted on an LB-Miller agar plate with or without 50 µM putrescine. Plates were incubated at 37°C and swarming diameters were measured at the indicated time. Experiments were performed in biological triplicate. Data are presented as mean ± SD are shown. (**A**) Swarming diameter of *P. mirabilis* PM7002 (WT) and HO110 (Δ*potE*) on an LB-Miller agar plate without putrescine. White circles and gray circles indicate the swarming diameters of WT and HO110, respectively. Statistical significance of the difference in swarming diameter at 21 h was assessed using student’s *t*-test. (**B**) Swarming diameter of *P. mirabilis* PM7002 (WT), HO118 (Δ*sapBCDF*), HO119 (Δ*pstSCAB*), and KY4 (Δ*sapBCDF* Δ*pstSCAB*) on LB-Miller agar plate without putrescine. White circles, white squares, white triangles, and white diamonds indicate the swarming diameters of WT, HO118 (Δ*sapBCDF*), HO119 (Δ*pstSCAB*), and KY4 (Δ*sapBCDF* Δ*pstSCAB*), respectively. Swarming diameters at 21 h were compared using one-way ANOVA followed by Tukey’s test. Different letters indicate significantly different means (*P* < 0.01). (***C***) Swarming diameter of *P. mirabilis* TF13 (pSK710/PM7002), TF17 (pSK710-*pstS*^+^*C*^+^*A*^+^*B*^+^/HO119), and TF24 (pSK710/HO119) on an LB-Miller agar plate without putrescine. White circles, white squares, and white triangles indicate the swarming diameter of TF13, TF17, and TF24, respectively. Swarming diameters at 21 h were compared using one-way ANOVA followed by Tukey’s test. Different letters indicate significantly different means (*P* < 0.01). (**D**) Swarming diameter of *P. mirabilis* PM7002 (WT), HO118 (Δ*sapBCDF*), HO119 (Δ*pstSCAB*), and KY4 (Δ*sapBCDF* Δ*pstSCAB*) on an LB-Miller agar plate with 50 µM putrescine. Black circles, black squares, black triangles, and black diamonds indicate the swarming diameter of WT, HO118 (Δ*sapBCDF*), HO119 (Δ*pstSCAB*), and KY4 (Δ*sapBCDF* Δ*pstSCAB*), respectively. Swarming diameters at 21 h were compared using one-way ANOVA followed by Tukey’s test. Different letters indicate significantly different means (*P* < 0.01). (**E**) Effect of putrescine supplementation on swarming diameter. White bars and black bars show the swarming diameter at 21 h without and with 50 µM putrescine supplementation, respectively. Statistical significance of the difference in swarming diameter was assessed using Student’s *t*-test (n.s., not significant, ^*^P < 0.05, ^***^P < 0.001).

* *Putrescine is a messenger molecule in swarming of *P. mirabilis* (13, 14). Swarming activity has been reported to decrease by deletion of *plaP*, a putrescine importer (14), although the effects of deletion of putrescine exporters on swarming remain unknown. Therefore, we evaluated swarming activity of PM7002 (WT), HO110 (Δ*potE*), HO118 (Δ*sapBCDF*), HO119 (Δ*pstSCAB*), and KY4 (Δ*sapBCDF* Δ*pstSCAB*). The swarming diameter increased in an incubation time-dependent manner, whereas there were no significant differences in swarming diameter between PM7002 (64 ± 8.5 mm) and HO110 (Δ*potE*) (77 ± 11 mm) after 19 h incubation (Fig. 5A). In contrast, the swarming diameter under putrescine depletion conditions was significantly decreased by the deletion of *pstSCAB* or *sapBCDF* (Fig. 5B). The swarming diameter of HO118 (Δ*sapBCDF*) was significantly lower than that of HO119 (Δ*pstSCAB*), and there is no significant difference between HO118 (Δ*sapBCDF*) and KY4 (Δ*sapBCDF* Δ*pstSCAB*) at 21 h incubation (Fig. 5B). Furthermore, we performed genetic complementation experiments. Unfortunately, introducing the chemically synthesized *sapBCDF* gene cloned in pSK710 into *Proteus mirabilis* was impossible, probably due to the toxicity of this gene when highly expressed. On the other hand, *pstSCAB*-complementation rescued the swarming activity of HO119. We confirm that *pstSCAB* contributes to swarming (Fig. 5C). Next, the effect of putrescine supplementation on swarming activity was evaluated to clarify whether the decrease in swarming activity was related to the reduction of putrescine in the culture medium, as caused by a decrease in putrescine export. Putrescine supplementation restored the swarming diameter of HO119 (Δ*pstSCAB*) to the same level as that of the wild-type (Fig. 5D and E). In HO118 (Δ*sapBCDF*) and KY4 (Δ*sapBCDF* Δ*pstSCAB*), putrescine supplementation significantly increased the swarming diameter (Fig. 5E). These results indicate that putrescine export by SapBCDF and PstSCAB is important for normal swarming activity, and that putrescine export activity influences swarming activity (Fig. 3B and 5E).

**Fig 6:**
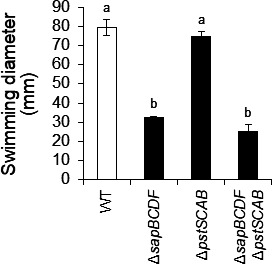
Swimming activity of *P. mirabilis* WT, Δ*pstSCAB*, Δ*sapBCDF*, and Δ*pstSCAB* Δ*sapBCDF. P. mirabilis* PM7002 (WT), HO110 (Δ*potE*) HO118 (Δ*sapBCDF*), HO119 (Δ*pstSCAB*), and KY4 (Δ*sapBCDF* Δ*pstSCAB*) were grown overnight in an LB-Miller broth. Two microliters of overnight cultures were spotted on an LB-Miller agar plate. Plates were incubated at 37°C for 13 h, and then swimming diameters were measured. Experiments were performed in biological triplicate and mean ± SD are shown. Swimming diameters at 13 h were compared using one-way ANOVA followed by Tukey’s test. Different letters indicate significantly different means (*P* < 0.01).

 *P. mirabilis* shows swimming motility in addition to swarming. The contribution of *pstSCAB* and *sapBCDF* to swimming activity was evaluated. There were no significant differences between swimming diameter of WT and HO119 (Δ*pstSCAB*), on the other hand, the swimming diameter of HO118 (Δ*sapBCDF*) and KY4 (Δ*sapBCDF* Δ*pstSCAB*) were significantly decreased compared to that of the WT (Fig. 6; Fig. S8).

## DISCUSSION

 Putrescine is essential for motility and urinary tract colonization (13, 35). A previous study showed that PlaP acts as a primary putrescine importer and an essential protein in putrescine-mediated communication (14). Additionally, using T44, a putrescine analog, to inhibit putrescine uptake, reduced urothelial cell invasion by *P. mirabilis* (14). These studies demonstrate that the inhibition of putrescine-mediated cell-cell communication is a promising new approach to prevent *P. mirabilis* UTIs. However, no putrescine export proteins have been reported to date. In this study, we generated a *P. mirabilis* transposon mutant library and screened PstSCAB as a putrescine exporter candidate by measuring the putrescine concentrations in culture supernatants using our previously developed high-throughput putrescine quantification method (23). Analysis of the gene deletion mutants and S.I.Arg assays showed that PstSCAB and SapBCDF are putrescine exporters in *P. mirabilis* PM7002.

 PstSCAB is a high-affinity phosphate transporter found in some bacteria (36–38). In *P. mirabilis*, PstS and PstA are necessary for the colonization of the host bladder and kidney (32) and biofilm formation in the urinary tract (39). Therefore, PstSCAB has been reported as a virulence factor in *P. mirabilis*. However, biochemical analysis of PstSCAB in *P. mirabilis* has not yet been performed. We carried out a phosphate‐uptake assay in LB-Miller medium; in the wild type, the phosphate in the culture supernatants was decreased, whereas no such decrease was observed in Δ*pstSCAB* (Fig. 1B), thereby confirming that PstSCAB functions as a phosphate transporter in *P. mirabilis*. In *P. mirabilis* HI4320, similar levels of constitutive alkaline phosphatase expression have been observed in Δ*pstS* and Δ*pstA* mutants in LB-Miller medium, human urine, and phosphate-limiting medium (32). However, *in vitro* competition assays by co-culturing WT with Δ*pstS* or Δ*pstA* mutants showed the following: compared with the WT, the survival rate of Δ*pstS* was reduced in the LB-Miller medium, whereas the survival rate of Δ*pstA* was reduced in the phosphate-limiting minimal salt medium (32). These results suggest that the functions and/or expression patterns of PstSCAB vary depending on the growth environment. We used LB-Lennox and LB-Miller media, which have been used in previous studies as media with available phosphate content (32), to analyze *P. mirabilis* Δ*pstSCAB* mutants. Therefore, *P. mirabilis* Δ*pstSCAB* may show a different phenotype in phosphate-limited conditions. O’May et al. (39) performed comparative proteomic analyses between *P. mirabilis* HI4320 WT, Δ*pstS*, and Δ*pstA* during biofilm formation and discovered several protein candidates involved in biofilm formation. However, it was unclear why Δ*pstS* and Δ*pstA* had a weaker biofilm-forming activity than WT. Additionally, in *Pseudomonas aeruginosa* PAO1, PstS is involved in biofilm formation in a phosphate-independent manner (40). These previous reports suggest that another factor, besides phosphate, contributes to PstS-mediated biofilm formation. Polyamines have been reported to contribute to biofilm formation (41–43). Our results suggest that putrescine export via PstSCAB contributes to biofilm formation. However, further studies are needed to determine the relationship between putrescine export and biofilm formation by evaluating the biofilm formation ability of Δ*pstSCAB* and Δ*sapBCDF* strains with and without putrescine.

 Although SapABCDF has been identified as an antimicrobial peptide transporter in *Haemophilus influenzae* (44, 45) and *Salmonella* Typhimurium (46), we previously reported that SapBCDF is a putrescine exporter that functions under a neutral condition in *E. coli* (15). The identity of the proteins constituting the *sap* operon between *E. coli* and *P. mirabilis* is in the range of 68%‒79% (Table S3). In this study, we showed that SapBCDF, which has been reported as an antimicrobial peptide transporter in *P. mirabilis* (17), also functions as a putrescine exporter, as in *E. coli* (15).

 PotE is an ornithine-putrescine antiporter and contributes to putrescine excretion in *E. coli* in acidic environments (47). *Proteus mirabilis* possesses a PotE homolog with 81% identity (Table S3). However, putrescine concentrations in the culture supernatants of WT and HO110 (Δ*potE*) were similar (Fig. 2B). All the experiments in this study were performed under neutral pH conditions and a previous cohort study showed that patients with *P. mirabilis* UTIs have a neutral to alkaline urine (48–50). Hence, *P. mirabilis* shows virulence in an almost neutral environment, and unveiling the function of genes under neutral conditions is important. The finding that gene deletion of *potE* did not affect putrescine concentrations in culture supernatant suggests that, in *P. mirabilis* as in *E. coli*, *potE* is a gene expressed only under acidic conditions. Further analyses under acidic conditions are necessary to elucidate the detailed function of PotE in *P. mirabilis*.

 The swarming motility of HO118 (Δ*sapBCDF*), HO119 (Δ*pstSCAB*), and KY4 (Δ*sapBCDF* Δ*pstSCAB*) was significantly reduced compared to the WT (Fig. 5B). In the swimming assay, HO118 (Δ*sapBCDF*) and KY4 (Δ*sapBCDF* Δ*pstSCAB*) also showed a significant decrease in swimming activities, whereas HO119 (Δ*pstSCAB*) did not differ from the WT (Fig. 6). Regarding the difference in the contribution of PstSCAB between swarming and swimming, the contribution of putrescine to each type of motility may differ. Swarming is presumed to be a more coordinated movement than swimming, and precise signal transmission, such as putrescine-mediated communication, between cells is thought to be important. Consistent with this hypothesis, culture supernatants of HO119 (Δ*pstSCAB*) contained significantly higher levels of putrescine than those of HO118 (Δ*sapBCDF*) and KY4 (Δ*sapBCDF* Δ*pstSCAB*). We, therefore, propose that putrescine in HO119 is sufficient to maintain swimming activity at WT levels.

 There was still 4.4 ± 0.4 µM/OD_600_ of putrescine detected in the culture supernatants of KY4 (Δ*sapBCDF* Δ*pstSCAB*). LB-Lennox medium originally contains 1.46 µM putrescine, corresponding to 0.27 µM/OD_600_ in KY4, suggesting that other putrescine excretion mechanisms or exporters are present in *P. mirabilis*. The putrescine level in the culture supernatants of KY4 (Δ*sapBCDF* Δ*pstSCAB*) was significantly lower than that of HO119 (Δ*pstSCAB*) but not significantly different from that of HO118 (Δ*sapBCDF*) (Fig. 3B). We demonstrated that SapBCDF and PstSCAB are putrescine exporters under the conditions used in the experiment. The effect of gene deletion on the putrescine concentration in the culture supernatant was more significant for SapBCDF than for PstSCAB (Fig. 3B, 4B and C). These results suggest the possibility that unknown putrescine exporters serve as alternatives to PstSCAB when PstSCAB is inactivated. Identification of these unknown putrescine excretion mechanisms and putrescine exporters is important for understanding the pathogenicity and putrescine-mediated cell-cell communication in *P. mirabilis*.

 Our results revealed that PstSCAB is a phosphate transporter (Fig. 1B) and suggested that it is involved in regulating intracellular phosphate concentrations in *P. mirabilis*. This finding leaves open the possibility that a deficiency in intracellular phosphate by *pstSCAB* deletion indirectly reduces putrescine export by SapBCDF. The possibility of regulation of putrescine exporter activity of SapBCDF through control of intracellular phosphate concentration by PstSCAB will be verified in the future.

## Data Availability

All data pertinent to this work are contained within this article or available on request from the corresponding author.

## SUPPLEMENTAL MATERIAL

Supplemental Figures spectrum.04306-23-s0001.pdf Supplemental Figures without highlight

Supplemental Tables spectrum.04306-23-s0002.docx Supplemental Tables S1 to S3 without highlight.

ASM does not own the copyrights to Supplemental Material that may be linked to, or accessed through, an article. The authors have granted ASM a non-exclusive, world-wide license to publish the Supplemental Material files. Please contact the corresponding author directly for reuse.
