# Supplementary material for: PstSCAB and SapBCDF are putrescine exporters in Proteus mirabilis
Source: Microbiol Spectr. 2025 Nov 14;14(1):e04306-23. doi: 10.1128/spectrum.04306-23 (PMC12772348; doi:10.1128/spectrum.04306-23)
Supplement: Supplemental Figures — without highlight [file spectrum.04306-23-s0001.pdf]

# Supporting information

## PstSCAB and SapBCDF are putrescine exporters in *Proteus mirabilis*

Yuta Sugiyama[1, †], Atsuo Nakamura[2], Hirokazu Ohta[1], Yuki Kontani[3], Hiromi Shimokawa[3], Rika Hirano[1, 3], Mikiyasu Sakanaka[1, ‡], Mitsuharu Matsumoto[2], and Shin Kurihara[1, 3, *]

[1] Faculty of Bioresources and Environmental Sciences, Ishikawa Prefectural University, Nonoichi, Ishikawa 921-8836, Japan
[2] Dairy Science and Technology Institute, Kyodo Milk Industry Co. Ltd, Tokyo 190-0182, Japan
[3] Faculty of Biology-Oriented Science and Technology, Kindai University, Kinokawa, Wakayama 649-6493, Japan

Present address
[†] Graduate School of Science and Technology, Gunma University, Kiryu, Gunma 376-8515, Japan
[‡] Faculty of Agriculture, Ryukoku University, Otsu, Shiga 520-2194, Japan

[*]Corresponding author: skurihara@waka.kindai.ac.jp

**Figure S1.** Putrescine concentrations in the culture supernatants of candidates of putrescine exporter mutants.

**Figure S2.** HPLC analysis of culture supernatants of *Proteus mirabilis* PM7002.

**Figure S3.** Genotype of HO118 (Δ*sapBCDF*::*Cm*$^R$), HO119 (Δ*pstSCAB*::*Cm*$^R$), and KY4 (Δ*sapBCDF*::*Cm*$^R$ Δ*pstSCAB*::*Kan*$^R$).

**Figure S4.** Conversion of stable isotope-labeled arginine to stable isotope-labeled putrescine in *Proteus mirabilis* PM7002

**Figure S5.** Putrescine production of *Proteus mirabilis* PM437 (Δ*speA*).

**Figure S6.** GC-MS analysis of stable isotope-labeled putrescine.

**Figure S7.** Effects of arginine on the growth of *Proteus mirabilis* PM7002.

**Figure S8.** Swimming of *Proteus mirabilis* PM7002 and HO118 (Δ*sapBCDF*::*Cm*$^R$), HO119 (Δ*pstSCAB*::*Cm*$^R$), and KY4 (Δ*sapBCDF*::*Cm*$^R$ Δ*pstSCAB*::*Kan*$^R$).

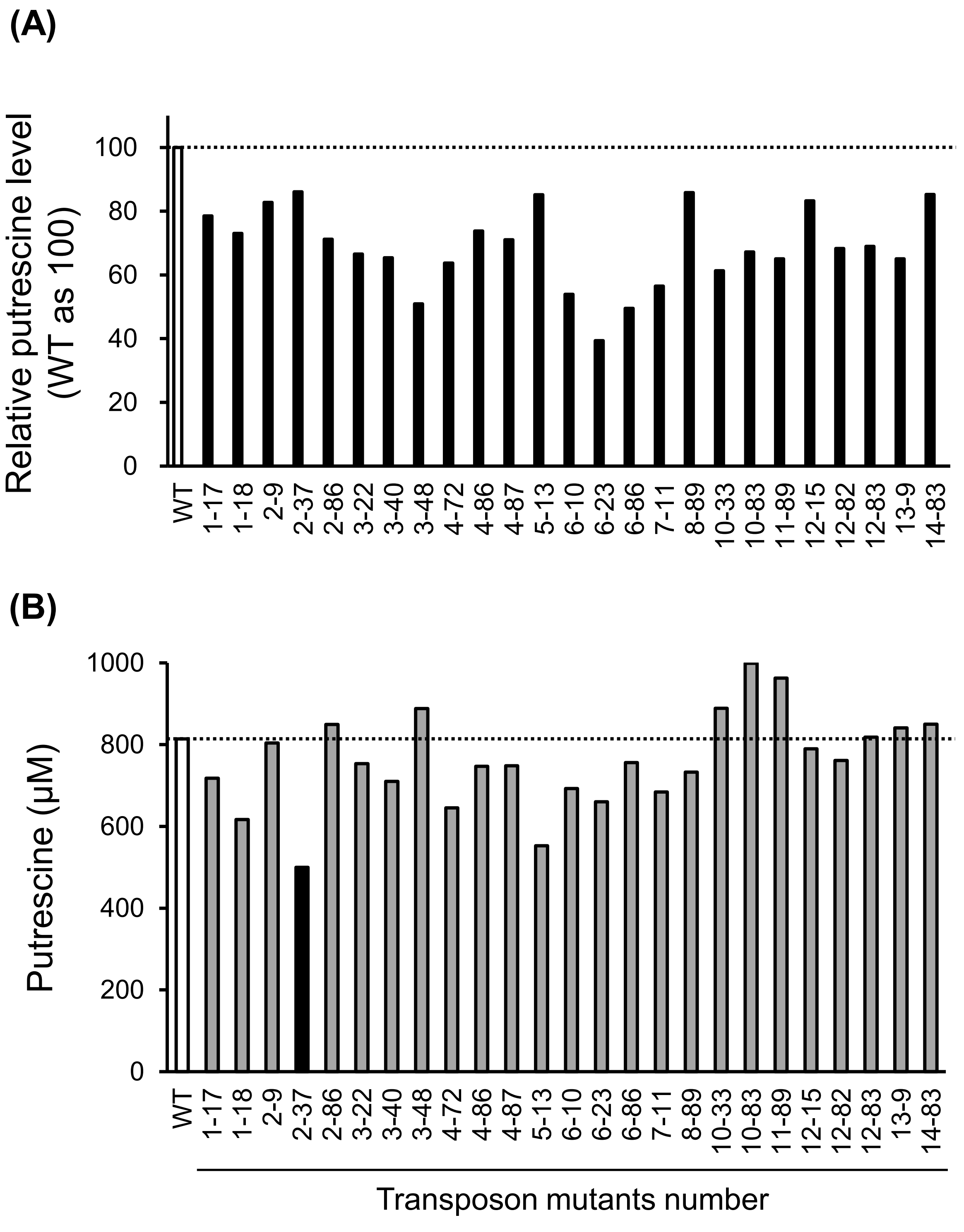


**Figure S1. Putrescine concentrations in the culture supernatants of candidates of putrescine exporter mutants.**

(A) Relative putrescine levels of the candidates of putrescine exporter gene mutants and measured by PuO-POD-4AA-TOPS method (*Anal Biochem*., 593:113607, 2020). Putrescine concentration of wild-type (WT) was set as 100, and putrescine concentration of each mutant relative to WT are shown.

(B) The putrescine concentration in the culture supernatants of the candidates of putrescine exporter gene mutants measured by HPLC.

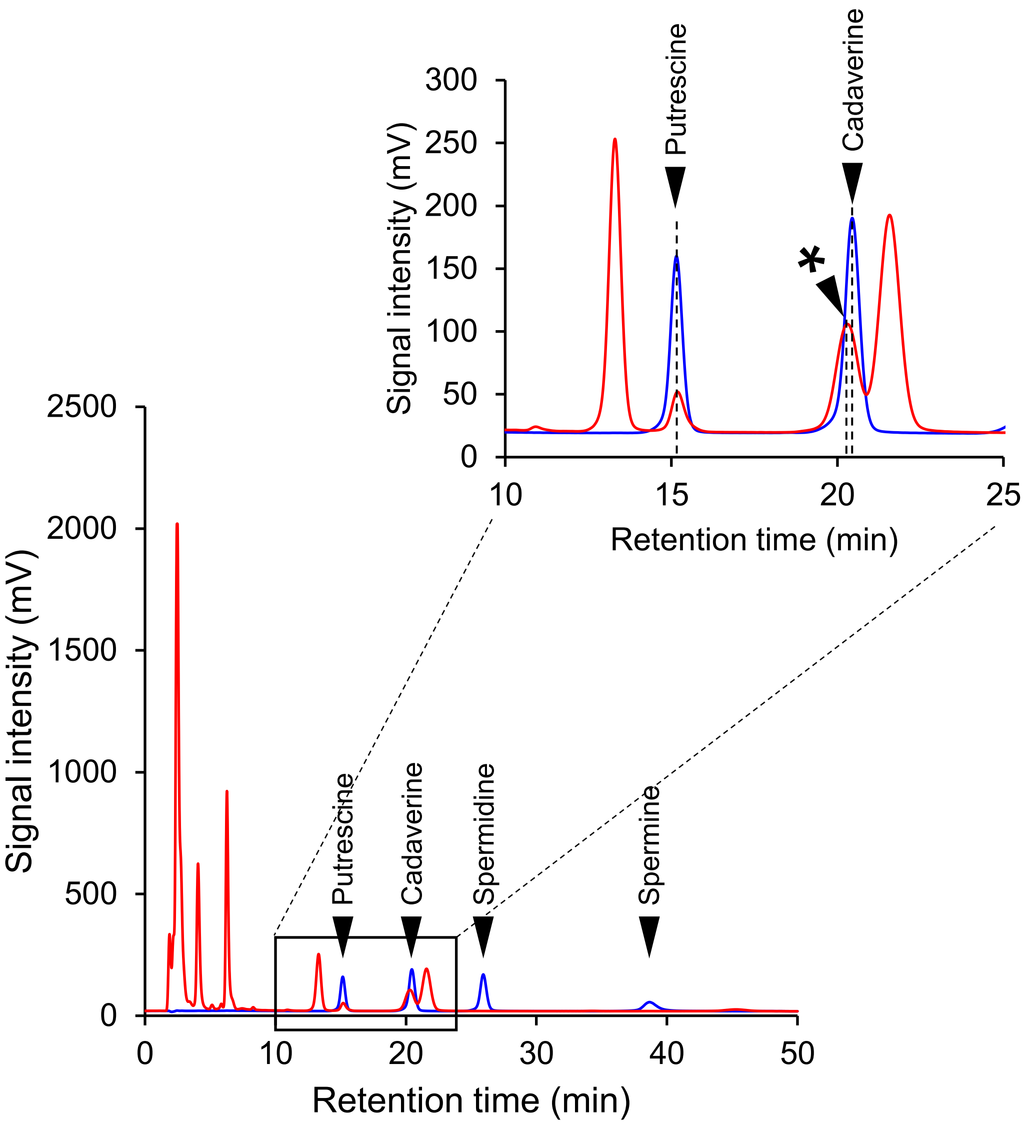


**Figure S2. HPLC analysis of culture supernatants of *Proteus mirabilis* PM7002.**

HPLC chromatograms of 100 µM polyamine standard (blue) and the 10-fold diluted culture supernatants of *P. mirabilis* PM7002 wild-type (red) cultivation in LB-Miller medium. The inset shows an expanded view of the boxed region between 10 to 25 min. * indicates an unidentified amine with retention time close to cadaverine.

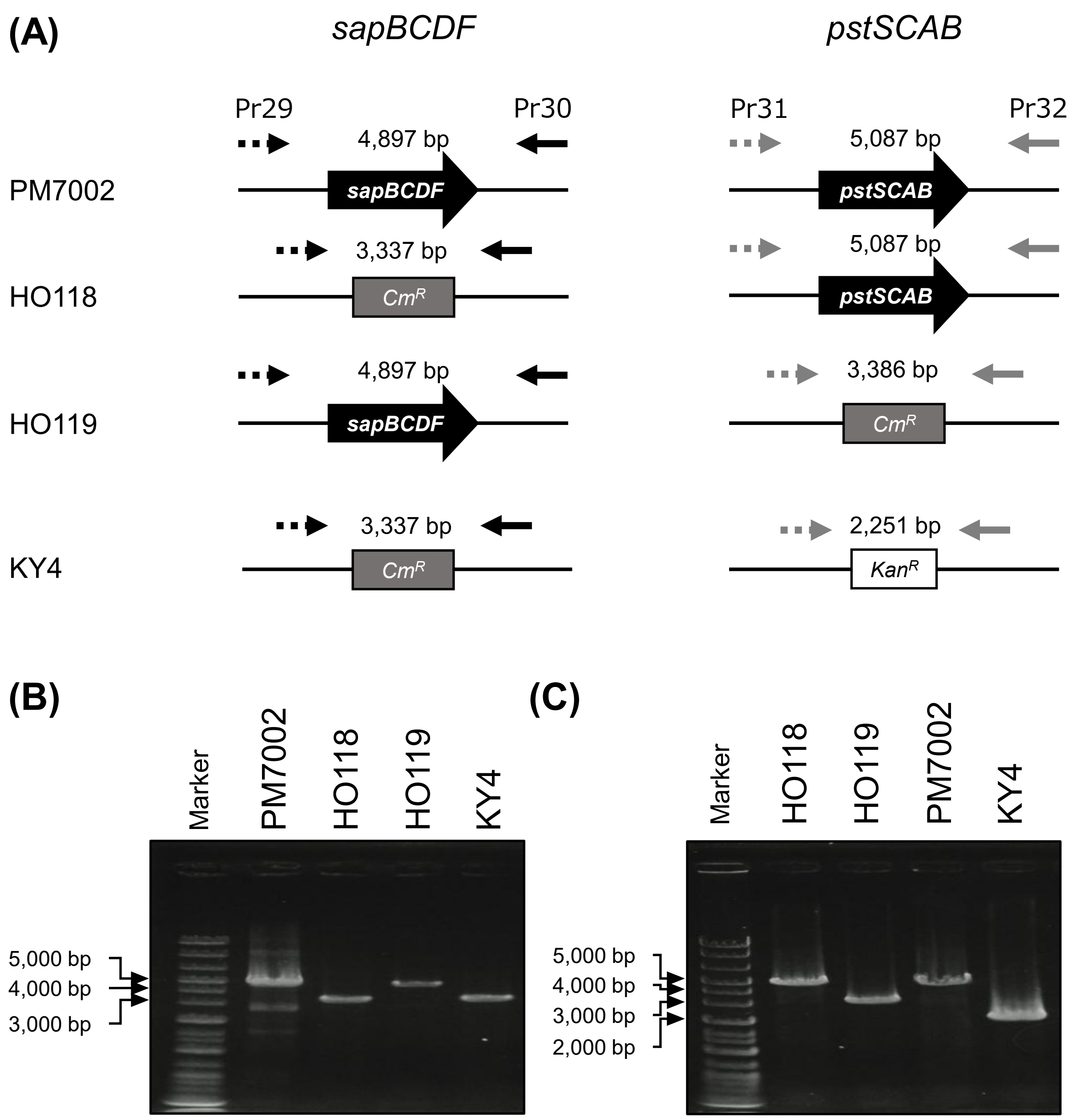


**Figure S3. Genotype of HO118 (Δ*sapBCDF*::*Cm*$^R$), HO119 (Δ*pstSCAB*::*Cm*$^R$), and KY4 (Δ*sapBCDF*::*Cm*$^R$ Δ*pstSCAB*::*Kan*$^R$).**

(A) Genome structures of *sap* and *pst* operon in PM7002 (wild-type), HO118, HO119, and YK4. Dashed and gray arrows indicate the primers used for genotype analysis of *sap* and *pst* operon, respectively. See also Table S1 and S2.
(B) Confirmation of *sapBCDF* to *Cm*$^R$ replacement.
(C) Confirmation of *pstSCAB* to *Cm*$^R$ or *Kan*$^R$ replacement.
Gene Ladder Wide 1 (Nippon Gene, Tokyo, Japan) was used as DNA size marker.

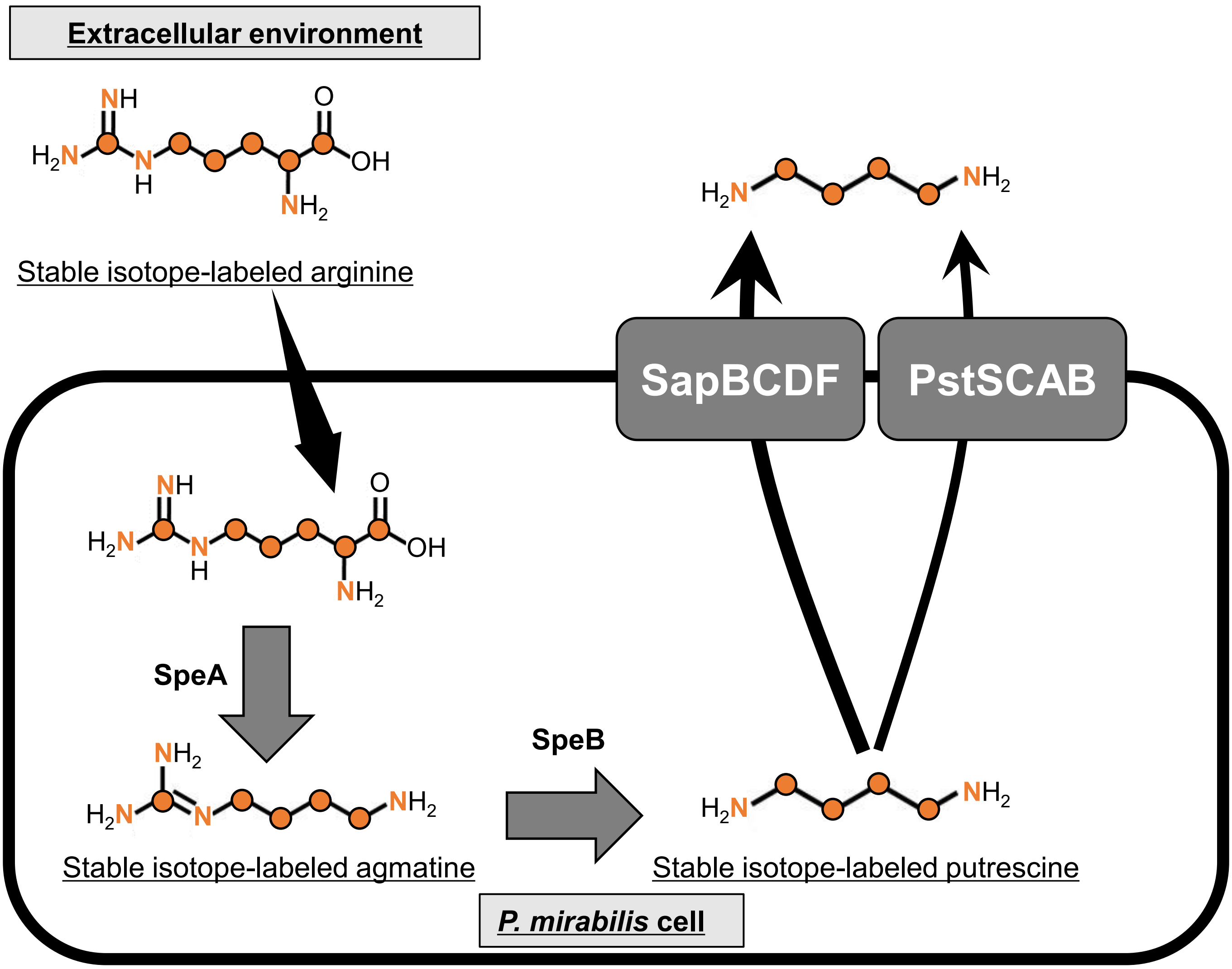


**Figure S4. Conversion of stable isotope-labeled arginine to stable isotope-labeled putrescine in *Proteus mirabilis* PM7002**.

Orange circle and orange characters indicate stable isotope-labeled atoms. Stable isotope-labeled putrescine in the culture supernatants were measured.

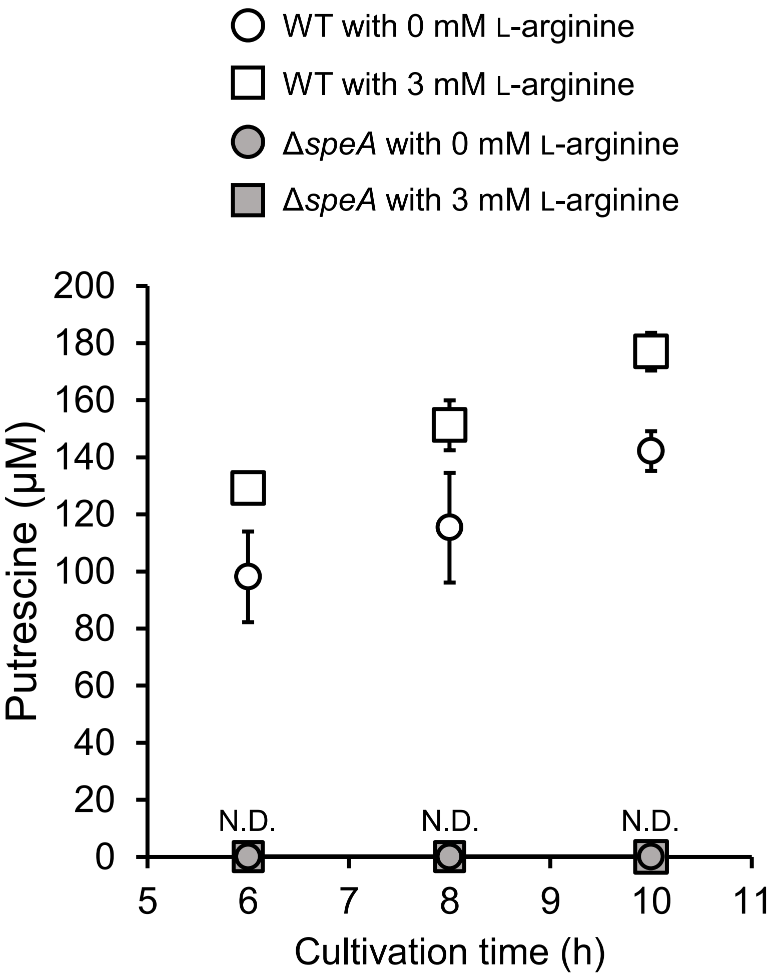


**Figure S5. Putrescine production of *Proteus mirabilis* PM437 (Δ*speA*)**.

*P. mirabilis* PM7002 WT and PM437 (Δ*speA*) were grown in LB-Lennox medium containing 0 or 3 mM L-arginine at 37 ˚C. The putrescine concentrations in the culture supernatants were measured at the indicated time points. These experiments were performed in biological triplicate and means ± SD are shown.

N.D., not detected.

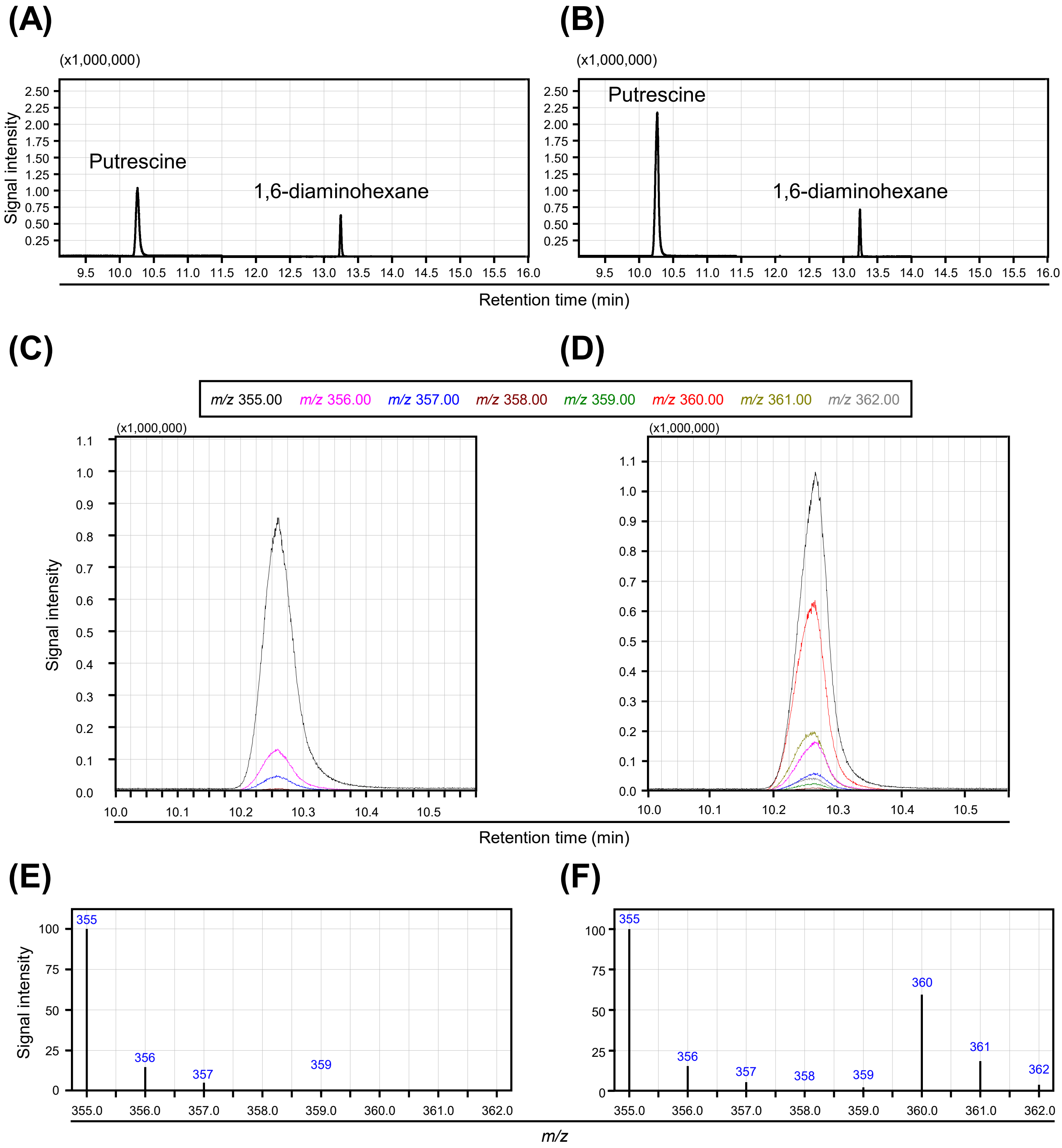


**Figure S6. GC-MS analysis of stable isotope-labeled putrescine**.

Stable-isotope labeled putrescine (S.I.Put) in the culture supernatants were measured using GC-MS. 1,6-diaminohexane was used as an internal standard.

(A) Summed selected ion monitoring (SIM) chromatogram (*m/z* 355–362) of 40 μM putrescine standard.

(B) Summed SIM chromatogram (*m/z* 355–362) of putrescine in culture supernatant from *Proteus mirabilis* PM7002 wild-type after 8 h cultivation in M9+Tryptone medium containing 3 mM stable isotope-labeled arginine (S.I.Arg).

(C) Observed SIM chromatogram of 40 μM putrescine standard.

(D) Observed SIM chromatogram of putrescine in the culture supernatant from *P. mirabilis* PM7002 wild-type after 8 h cultivation in M9+Tryptone medium containing 3 mM S.I.Arg. The signal at *m/z* 360 (red line) is S.I.Put, which originates from S.I.Arg (see Figure S4).

(E) Mass spectrum of 40 μM putrescine standard.

(F) Mass spectrum of putrescine in the culture supernatant from *P. mirabilis* PM7002 wild-type after 8 h cultivation in M9+Tryptone medium containing 3 mM S.I.Arg.

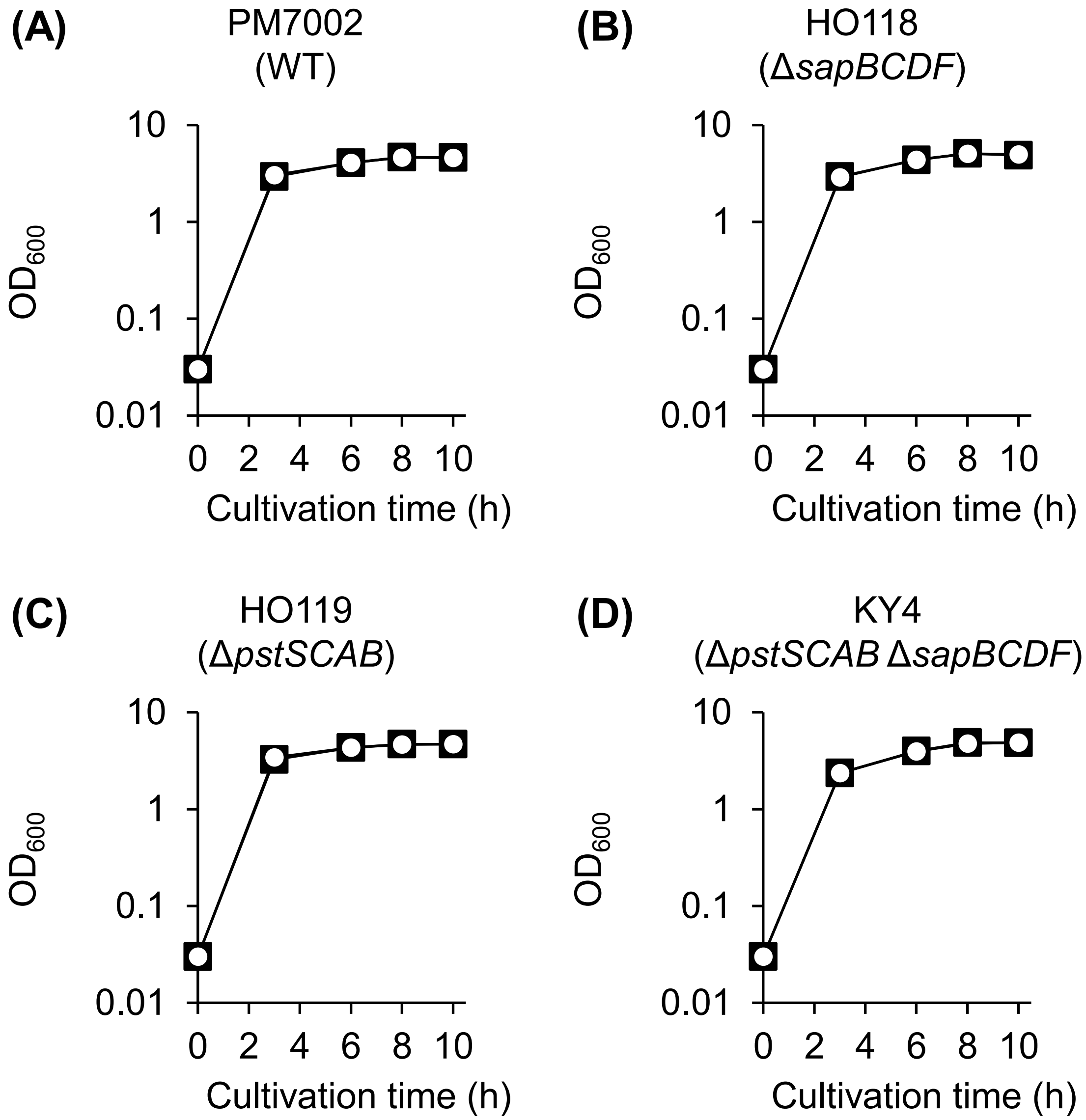


**Figure S7. Effects of arginine on the growth of *Proteus mirabilis* PM7002**.

*Proteus mirabilis* PM7002 (A), HO118 (B), HO119 (C), and KY4 (D) were grown in M9+Tryptone medium containing 0 or 3 mM stable isotope-labeled L-arginine, and $OD_{600}$ was measured at the indicated time. These experiments were performed in biological triplicate and means ± SD are shown.

White circles and black squares represent the mean of the $OD_{600}$ of 0 or 3 mM stable isotope-labeled L-arginine supplemented condition, respectively.

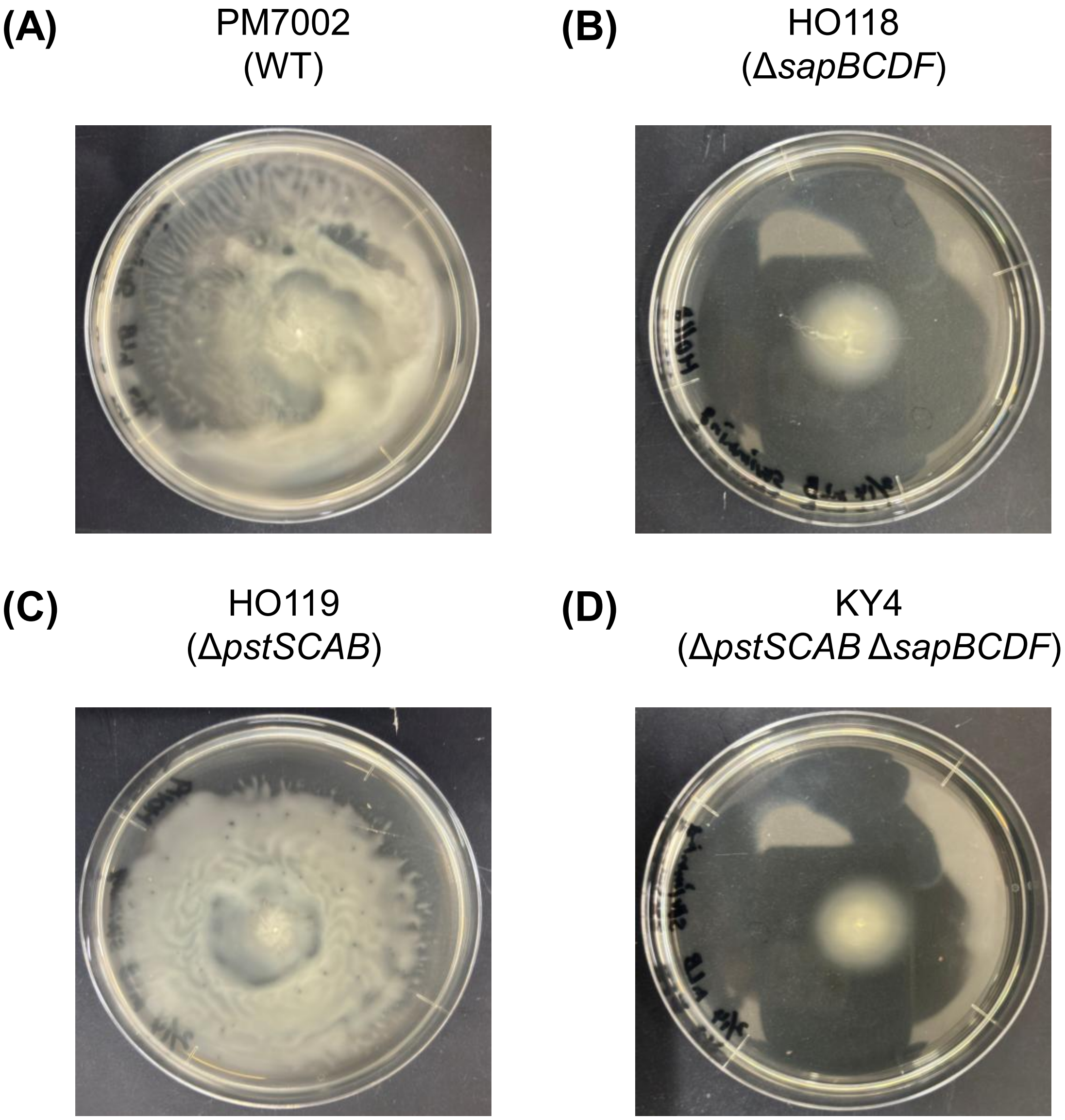


**Figure S8. Swimming of *Proteus mirabilis* PM7002 and HO118 (Δ*sapBCDF*::*Cm*$^R$), HO119 (Δ*pstSCAB*::*Cm*$^R$), and KY4 (Δ*sapBCDF*::*Cm*$^R$ Δ*pstSCAB*::*Kan*$^R$).**

*Proteus mirabilis* PM7002 (A), HO118 (B), HO119 (C), and KY4 (D) were spotted on LB-Miller plate (agar 0.25%) and incubated for 13 h at 37˚C. These experiments were performed in biological triplicate and the representative photographs are shown.
