# Supplementary material for: PstSCAB and SapBCDF are putrescine exporters in Proteus mirabilis
Source: Microbiol Spectr. 2025 Nov 14;14(1):e04306-23. doi: 10.1128/spectrum.04306-23 (PMC12772348; doi:10.1128/spectrum.04306-23)
Supplement: Supplemental Tables — S1 to S3 without highlight. [file spectrum.04306-23-s0002.docx]

**Supplementary Tables**

PstSCAB and SapBCDF are putrescine exporters in *Proteus mirabilis*

Yuta Sugiyama^1, †^, Atsuo Nakamura^2^, Hirokazu Ohta^1^, Yuki Kontani^3^, Hiromi Shimokawa^3^, Rika Hirano^1, 3^, Mikiyasu Sakanaka^1, ‡^, Mitsuharu Matsumoto^2^,

and Shin Kurihara^1, 3, *^

^1^Faculty of Bioresources and Environmental Sciences, Ishikawa Prefectural University,

　Nonoichi, Ishikawa 921-8836, Japan

^2^Dairy Science and Technology Institute, Kyodo Milk Industry Co. Ltd,

　Tokyo 190-0182, Japan

^3^Faculty of Biology-Oriented Science and Technology, Kindai University, Kinokawa,

　Wakayama 649-6493, Japan

Present address

^†^Graduate School of Science and Technology, Gunma University, Kiryu, Gunma 376-8515, Japan

^‡^Faculty of Agriculture, Ryukoku University, Otsu, Shiga 520-2194, Japan

^*^Corresponding author: skurihara@waka.kindai.ac.jp

**Table S1. Primers used in this study.**

| **Primer**  **No.** | **Primer name** | **Sequence** |
| --- | --- | --- |
| Pr1 | EZ-ProteusGenome_1st | GGCCACGCGTCGACTAGTACNNNNNNNNNNGTAC |
| Pr2 | EZ_KanPCR3_rv | GCATAAATTCCGTCAGCCAG |
| Pr3 | EZ_KanPCR_fw | GATGTTGGACGAGTCGGAAT |
| Pr4 | miniTn5-ProteusGenome_2nd | GGCCACGCGTCGACTAGTAC |
| Pr5 | EZ_KanPCR_rv | ATCGGGCTTCCCATACAATC |
| Pr6 | EZ_KanSeq_fw | CTCGGTGAGTTTTCTCCTTC |
| Pr7 | EZ_KanSeq_rv | TCGCGAGCCCATTTATACCC |
| Pr8 | EZ-KanSeq2_fw | GCAGTTTCATTTGATGCTCG |
| Pr9 | pstSCAB_up_fw | **GGTCGACGGATCCCC**TGCATCTTCACGATAGACTC |
| Pr10 | pstSCAB_upS_rv | ACGGTGTGCCACTCATAACGAGGCAAATCATGGAT |
| Pr11 | pstSCAB_downS_fw | GGCAGTGTTGAGCAGAATCCCTCCTGTGGGATAGG |
| Pr12 | pstSCAB_down_rv | **TGCATCCGCGGGCCC**CACACGCTACGGACATGGTT |
| Pr13 | sapBCDF_up_fw | **GGTCGACGGATCCCC**TATAATCCCAGCCCGTTAAA |
| Pr14 | sapBCDF_upS_rv | ACGGTGTGCCACTCAGGGTTTGTTGTAGCCTCTTG |
| Pr15 | sapBCDF_downS_fw | GGCAGTGTTGAGCAGTATTAAACAGCATTAACATC |
| Pr16 | sapBCDF_down_rv | **TGCATCCGCGGGCCC**CAGCAGGTGCGAAACCGATA |
| Pr17 | potE_up_fw | **GGTCGACGGATCCCC**TTCCATCAGTATATCATGCC |
| Pr18 | potE_upS_rv | ACGGTGTGCCACTCACGTTTTACTCTCCGGCTGAC |
| Pr19 | potE_downS_fw | GGCAGTGTTGAGCAGCACACGCTATTCGTTATATT |
| Pr20 | potE_down_rv | **TGCATCCGCGGGCCC**CTTACTAAGCATCCTCGTTG |
| Pr21 | pSK-Cmr2_fw | TGAGTGGCACACCGTTTCTG |
| Pr22 | pSK-Cmr2_rv | CTGCTCAACACTGCCAACTT |
| Pr23 | KmR_fw | CTGTCTCTTATACACATCTC |
| Pr24 | KmR_rv | TTAGAAAAACTCATCGAGCATC |
| Pr25 | pstSCAB_up_KnR15_rv | GTGTATAAGAGACAGTAACGAGGCAAATCATGGAT |
| Pr26 | pstSCAB_down_KnR15_fw | GATGAGTTTTTCTAATCCCTCCTGTGGGATAGGTAG |
| Pr27 | KmR_L_fw | CTGTCTCTTATACACATCTCAACCATCATCGATG |
| Pr28 | pstSCAB_down_S2_rv | **TGCATCCGCGGGCCC**GTAATCAAAATGAAGAATAT |
| Pr29 | proteus_dsapBCDF_check2_fw | TCGTGCCTCTTGGGCTTATG |
| Pr30 | sapBCDF_PCR_rv | GGATTTAACATTGCACGGCT |
| Pr31 | proteus_dpstSCAB_check2_fw | CATGACCCTTGACGTAGTAG |
| Pr32 | pstSCAB_PCR_rv | GAAGTGATCTGCGCTGAATC |

*^a^*Bold characters indicate the nucleotide sequences used for In-Fusion cloning (Takara Bio).

**Table S2. Primer pairs and purpose.**

| **Primer pair** | **Purpose** |
| --- | --- |
| Pr1/Pr2 | Amplification of upstream of *Kan^R^* insertion locus using semi-degenerate primer |
| Pr1/Pr3 | Amplification of downstream of *Kan^R^* insertion locus using semi-degenerate primer |
| Pr4/Pr5 | Amplification of upstream of *Kan^R^* insertion locus containing 9 bases of genomic sequence |
| Pr4/Pr6 | Amplification of downstream of *Kan^R^* insertion locus containing 9 bases of genomic sequence |
| Pr9/Pr10 | Amplification of 500 bp upstream from start codon of *pstSCAB* |
| Pr11/Pr12 | Amplification of 500 bp downstream from stop codon of *pstSCAB* |
| Pr13/Pr14 | Amplification of 500 bp upstream from start codon of *sapBCDF* |
| Pr15/Pr16 | Amplification of 500 bp downstream from stop codon of *sapBCDF* |
| Pr17/Pr18 | Amplification of 500 bp upstream from start codon of *potE* |
| Pr19/Pr20 | Amplification of 500 bp downstream from stop codon of *potE* |
| Pr21/Pr22 | Amplification of *Cm^R^* from pKNG101 |
| Pr23/Pr24 | Amplification of *Kan^R^* from genomic DNA of the transposon mutant |
| Pr9/Pr25 | Amplification of 515 bp of DNA fragments contain 500 bp upstream from start codon of *pstSCAB* and 15 bp of complementary sequence of *Kan^R^* gene. |
| Pr12/Pr26 | Amplification of 515 bp of DNA fragments contain 500 bp downstream from stop codon of *pstSCAB* and 15 bp of complementary sequence of *Kan^R^* gene. |
| Pr27/Pr28 | Ligation of *Kan^R^* gene and 515 bp of DNA fragments contain 500 bp downstream from stop codon of *pstSCAB* and 15 bp of complementary sequence of *Kan^R^* gene by PCR. |
| Pr29/Pr30 | Confirmation of *sapBCDF* deletion |
| Pr31/Pr32 | Confirmation of *pstSCAB* deletion |

**Table S3. Similarity of PstSCAB, SapBCDF, and PotE between *P. mirabilis* and *E. coli*.**

|  | *P. mirabilis* | *E. coli* | | *Haemophilus influenzae* | | *Salmonella enterica*  serovar. typhimurium | |
| --- | --- | --- | --- | --- | --- | --- | --- |
|  | Uniprot  entry No. | Uniprot  entry No. | Similarity (%)  to *P. mirabilis* | Uniprot  entry No. | Similarity (%)  to *P. mirabilis* | Uniprot  entry No. | Similarity (%)  to *P. mirabilis* |
| PstS | B4EZJ5 | P0AG82 | 77 | P45192 | 50 | Q8ZKX5 | 78 |
| PstC | B4EZJ6 | P0AGH8 | 85 | P45191 | 54 | Q7CPE6 | 86 |
| PstA | B4EZJ7 | P07654 | 81 | P45190 | 57 | Q8ZKX6 | 81 |
| PstB | B4EZJ8 | P0AAH0 | 91 | Q4QK92 | 77 | P63365 | 91 |
| SapB | B4EWK9 | P0AGH3 | 71 | P45286 | 33 | P0A2J3 | 70 |
| SapC | B4EWL0 | P0AGH5 | 68 | P45287 | 44 | P0A2J5 | 68 |
| SapD | B4EWL1 | P0AAH4 | 79 | P45288 | 61 | P36636 | 78 |
| SapF | B4EWL2 | P0AAH8 | 72 | P45289 | 49 | P36638 | 72 |
| PotE | B4EUP1 | P0AAF1 | 81 | P44768 | 72 | Q8ZQW7 | 80 |

Their sequences are obtained from Uniprot (https://www.uniprot.org/).

Similarity (%) is from the alignment results using ClustalW (https://www.genome.jp/tools-bin/clustalw).
